# Digital Anterior Segment Photography in Clinical Practice: Mixed Methods Study

**DOI:** 10.2196/89441

**Published:** 2026-09-04

**Authors:** Vidhi Naik, Yarrow Scantling-Birch, Emma Jones, Rishi Ramessur

**Affiliations:** 1 Aberdeen Royal Infirmary, Aberdeen, United Kingdom; 2 Moorfields Eye Hospital NHS Foundation Trust, London, United Kingdom; 3 School of Informatics, University of Edinburgh, Room 3.42, Informatics Forum, 10 Crichton Street, Edinburgh, EH8 9AB, United Kingdom, +44 131 651 5661

**Keywords:** anterior eye segment, smartphone, photography, telemedicine, keratitis, diagnostic imaging

## Abstract

**Background:**

In the digital era, smartphone technology has significantly advanced ocular imaging, allowing for high-resolution anterior segment photography (ASP) to enhance referrals, decision-making, and disease monitoring within clinical workflows.

**Objective:**

This exploratory quality-improvement project aims to examine clinician experiences with 4 smartphone-based and slit-lamp–based ASP workflows used in eye casualty and corneal services.

**Methods:**

A mixed methods project was conducted at a tertiary eye hospital in England (United Kingdom) between October 21, 2024, and December 6, 2024. The full imaging setups of 4 ASP workflows were evaluated within an established clinical setting: the Magnifier app (a smartphone-based app; Apple Inc), QuikVue smartphone adapter (a smartphone-based camera lens attachment; VisuScience Meditech Co Ltd), NexYZ Slit-Lamp Adapter (a slit-lamp–based attachment; Celestron LLC), and Zeiss Slit Lamp Imaging Solution (ZSLIS; an integrated slit-lamp camera system; Carl Zeiss AG). Ophthalmology residents and corneal fellows completed nonvalidated usability surveys and participated in one-on-one interviews examining the role of ASP workflows in clinical practice. Survey data were analyzed using descriptive statistics. Interviews were transcribed and subjected to reflexive thematic analysis.

**Results:**

Among 25 survey respondents (17 residents and 8 fellows) and 12 interviewees, clinician-rated assessments of usability, cost-effectiveness, and clinical utility favored the Magnifier workflow, which received the highest ratings across these domains (19/25, 76%; 23/25, 92%; and 18/25, 72% strongly agreed, respectively). Five themes emerged from qualitative analysis: obstacles to clinical implementation, accessibility, quality and workflow, digital ethics, and innovation.

**Conclusions:**

No single ASP workflow proved superior in usability ratings and interview themes regarding clinical application. However, the use of the Magnifier workflow appeared to offer the most favorable balance of usability, cost-effectiveness, and clinical utility in clinician-rated assessments, though this reflects the full imaging workflow rather than the app alone. These findings are exploratory and require future work to examine workflow integration, data governance for smartphone imaging, and patient perspectives on ASP workflows.

## Introduction

Demand for ophthalmology services in the United Kingdom is rising with around 2.2 million people living with visual impairment [1]. An aging population drives this increase, with higher rates of ocular comorbidities and greater health care access requirements, thereby increasing workforce constraints [2]. Simultaneously, access to urgent and emergency eye care has expanded, with ophthalmic cases comprising 2% to 6% of emergency attendances [3]. Streamlining the patient pathway, particularly referrals, can ease service pressure. Evidence shows that including clinical photographs with referral histories improves diagnostic accuracy compared with written histories alone, potentially reducing the burden on Hospital Eye Services (HES) [4].

The anterior segment (AS) can be effectively imaged through anterior segment photography (ASP), which integrates the latest smartphone or smart tablet camera technology with the slit lamp. When combined with electronic patient records (EPRs), this surpasses clinical diagrams, enhances documentation, and allows for image-based triage. This allows for improved monitoring of challenging cases like infectious keratitis, which can be difficult to distinguish between infective organisms and support machine-learning models that allow for automated image-based screening and AI research [5].

Our project’s objective was to assess how 4 different ASP workflows—smartphone-based, slit-lamp–based, or integrated slit-lamp camera system—could be used among clinicians at a busy tertiary eye hospital.

## Methods

### Study Design and Setting

This exploratory mixed methods evaluation was cross-sectional, survey-based, and interview-based, designed to characterize clinician experience across 4 ASP workflows rather than test predefined hypotheses. The project ran from October 21 to December 6, 2024, at Moorfields Eye Hospital (MEH; City Road site, United Kingdom) eye casualty and outpatient cornea clinics. As an exploratory, single-center quality improvement evaluation, this project is intended to characterize clinician experience and generate hypotheses for future research, rather than provide definitive or generalizable comparisons between ASP workflows.

### ASP Workflows

Four standardized ASP workflows, each including distinct categories, were evaluated in this project in the eye casualty and corneal services. Workflows were defined and categorized, according to a recent scoping review, into smartphone-based devices (with or without attachments) and slit-lamp–based devices (slit-lamp cameras, conventional slit-lamps equipped with a digital camera, and slit-lamps equipped with a smartphone) [6]. Each device was integrated into a standard clinic workflow. A workflow was defined as the following standard of care: a patient was invited from the waiting room to an examination cubicle (estimated 12 m²), an appropriate history and examination were conducted after obtaining appropriate consent, the room light was turned off to maximize slit-lamp examination findings or set to ambient lighting (estimated 4000 K) to maximize smartphone-based examinations, and the specific ASP workflow of interest was assessed according to the manufacturer’s photo-capture instructions. These are described in further detail below.

**Figure 1.**
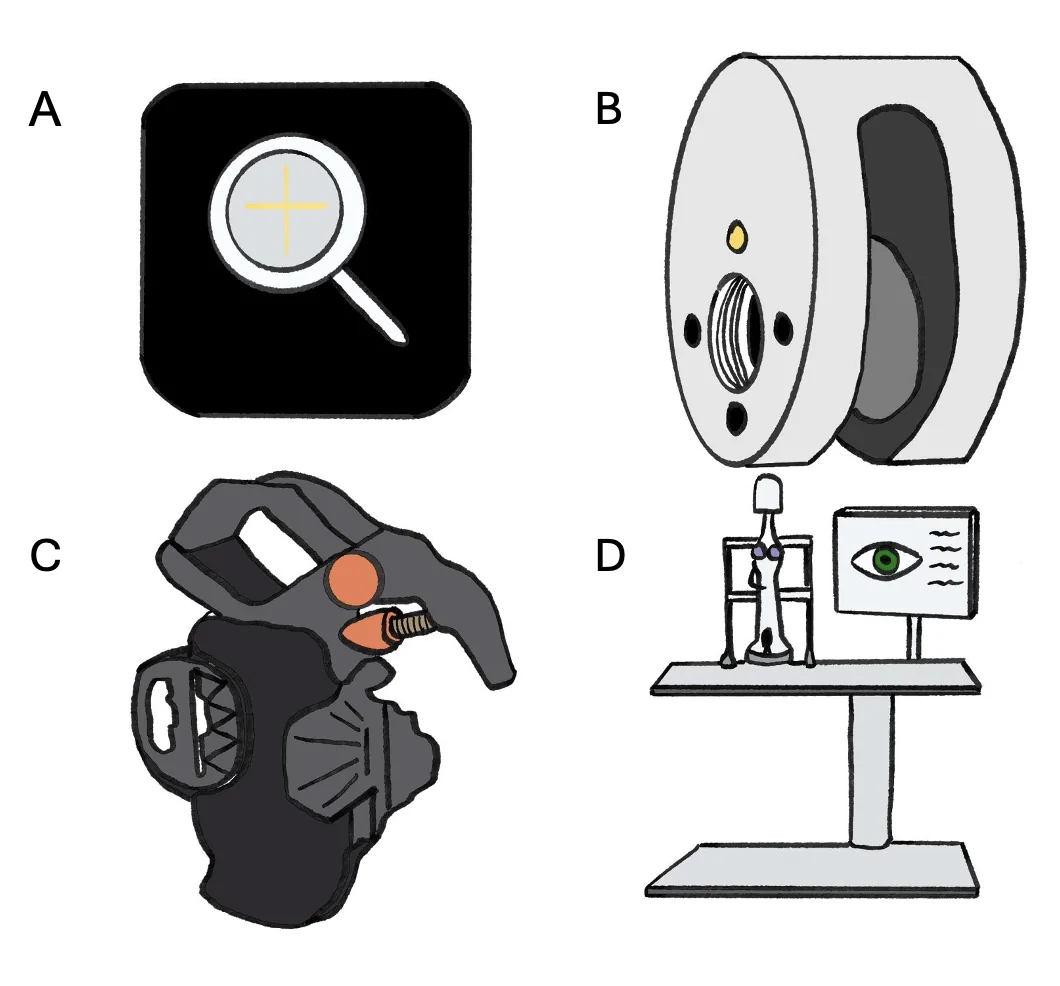
A sketch of the 4 anterior segment photography (ASP) workflows evaluated in this project. Illustrations were produced using the Procreate app (Savage Interactive): (A) Magnifier app, (B) QuikVue, (C) NexYZ, and (D) Zeiss Slit Lamp Imaging Solution.

The smartphone-based workflows comprised the Magnifier app (Apple Inc; Figure 1A), an iOS app running on an iPhone 13 Pro (Apple Inc) that isolates the ultrawide (macrocapable) lens to provide steady zoom and fixed focal length, and the QuikVue (VisuScience Meditech Co Ltd; Figure 1B), a macro lens attachment with built-in illumination and cobalt-blue filter, providing 10× magnification at a fixed focal length when coupled to a compatible smartphone [7,8].

The slit-lamp–based workflows comprised the NexYZ (Celestron LLC; Figure 1C), a universal smartphone adapter mounted to the slit-lamp eyepiece to enable image capture through slit-lamp optics, and the Zeiss Slit Lamp Imaging Solution (ZSLIS, Carl Zeiss AG; Figure 1D), an integrated slit-lamp camera system producing high-resolution images via a dedicated optical and camera assembly [9,10]. The NexYZ workflow was assessed by coupling a smartphone to the adapter before mounting onto the slit-lamp (Haag-Streit BM 900), thereby using the optics of both the smartphone and the slit-lamp (diffuse illumination settings: 10× magnification, illumination angle 45°, 8×14 mm beam [height × width], and low illumination intensity with a diffused light source). The ZSLIS workflow was the only one that did not require a smartphone for image capture.

In the case of smartphone-based workflows or slit-lamps equipped with a smartphone, a standardized iPhone 13 Pro (owned by a member of the project team with no ancillary accessories, such as a case) was used with the standard camera app native to iOS or the Magnifier app. The settings across ASP workflows changed only when applied to the Magnifier app, which isolates the ultrawide camera (12-megapixel, 13 mm lens, aperture f/1.8, and shutter speed 1/100 s). For all other workflows, the smartphone Camera app settings were set as follows, using the main telephoto camera (12-megapixel, 26 mm lens, aperture f/2.8, and shutter speed 1/100 s): fixed zoom (1×), fixed brightness and exposure at the automatic midpoint, no flash, auto-focus, and image-size exportation (maximum resolution, 4032×3024) [11].

Throughout this manuscript, references to a named ASP device (eg, the NexYZ) denote the complete clinical workflow in which it was evaluated. This includes, if applicable, the coupled smartphone, slit-lamp optics, room lighting, capture software with associated settings, and the image-handling pathway, rather than the isolated device. Outcomes reported reflect this full workflow and not the hardware component alone. For the purposes of readability, a named ASP device (eg, the NexYZ) may be used as shorthand for the complete workflow, not the isolated component.

### Ethical Considerations

This project was registered with and approved by the MEH Clinical Audit Department as a quality-improvement project (QIP; project number 1606). Formal National Health Service (NHS) Research Ethics Committee review was not sought because the project was classified locally as a QIP rather than research. This classification reflected that the project evaluated clinicians’ experience of ASP workflows already available or in use in standard clinical practice, with the primary purpose of informing local service improvement, workflow integration, documentation, and governance. The project did not allocate participants to an intervention, alter clinical care, or test a predefined hypothesis of clinical efficacy or diagnostic accuracy. This approach is consistent with UK Health Research Authority guidance distinguishing research from audit or service evaluation [12].

All clinician participants participated voluntarily and provided informed consent before survey participation and/or interview audio recording. For the illustrative clinical case images, patient consent was obtained in accordance with the MEH Patient Agreement to Media Recordings. This meant all patient-identifiable images were handled in line with the Data Protection Act 2018, the UK General Data Protection Regulation (GDPR), NHS England Bring Your Own Device (BYOD) guidance, and local governance policies [13,14]. Clinical images were captured on an approved iPhone 13 Pro using airplane mode to prevent transfer of images via cellular or unsecure networks. Captured images were immediately transferred to a secure NHS file server via a USB-linked file transfer to a clinical computer. Images were deleted immediately following transfer from the iPhone 13 Pro: no images were stored in the device’s general photo library or in an app-specific library, or transmitted via personal messaging or unsecure networks. This is all per the NHS England BYOD guidance and in keeping with local data protection policies. Survey and interview data were anonymized and deidentified before analysis. No participants received compensation for project participation.

### Project Recruitment

Corneal fellows and ophthalmology residents at MEH, City Road, were recruited to complete a survey and voluntary semistructured interviews. The recruitment process involved a project coordinator (VN) rotating through morning and afternoon sessions in the eye casualty and outpatient corneal clinics and approaching residents and fellows in these clinics about the QIP, with the available workflows present (excluding the ZSLIS); a total of 6 eye casualty sessions and 5 corneal clinics were visited during the period of October 21 to December 6, 2024.

All ophthalmology residents receive induction training for the ZSLIS prior to commencing a placement at MEH; therefore, familiarity with this system was assumed. All project participants had prior experience with smartphone-based ASP (defined as a general familiarity with smartphone photography for clinical ophthalmic assessment rather than the specific workflows tested), ensuring baseline familiarity. Prior experience with a tested ASP workflow was not an exclusion criterion, since the clinician’s perspective was still valuable information. Participants were further engaged through a hands-on familiarity exercise, lasting approximately 5 minutes and including practice in image capture and free exploration of each workflow’s features, prior to survey and interview administration.

To provide illustrative images and contextualize each ASP workflow within a diagnostic pathway, a single clinical case was used. A patient with corneal pathology attending the eye casualty was recruited with informed consent in accordance with the MEH Patient Agreement to Media Recordings. Images were captured by an ophthalmology resident (YS-B) using each of the 4 ASP workflows, including the same smartphone (iPhone 13 Pro) where applicable, during a single clinical encounter. Image acquisition was performed in either ambient lighting (estimated 4000 K) for smartphone-based examinations, except for the NexYZ which used slit-lamp optics, or with the room lights turned off for slit-lamp–based examinations. Two clinical examination cubicles were used; the ZSLIS required a separate cubicle as it used an integrated imaging system with a dedicated computer. Files from the ZSLIS were transferred to a secure NHS email account and stored on a secure NHS file server. For all other smartphone-based assessments, the native iOS camera app (iPhone 13 Pro) was used, except for the Magnifier workflow. Magnifier and camera settings were applied as described previously, and cubicle lighting and patient positioning were kept consistent between the 2 environments.

Participants were introduced to the project aims and given a short tutorial on each ASP workflow to standardize knowledge among clinicians. Surveys and interviews were designed to maximize user reflections on each ASP workflow and were not intended for formal hypothesis generation or statistical comparison. Each interview concluded with a discussion of differential diagnoses for a single clinical case, presented as the following vignette: a young male contact lens wearer presenting to eye casualty with a 24-hour history of eye pain and a corneal opacity on examination. Further discussion around global image characteristics across all 4 ASP workflows, including perceived functionality and costs, was held during interviews.

Formal sample size and power calculations were not conducted, reflecting the project’s exploratory design. Instead, target sample sizes were informed by prior literature and the practical limitations of recruiting within a niche specialty cohort. Twelve interviews were deemed sufficient for achieving thematic saturation, beyond which no new codes, subthemes, or themes are generated [15]. A sufficient benchmark for survey responses was deemed based on the limited number of residents at a single tertiary eye center, ranging between 20 and 30, and previous survey-based studies, among ophthalmology residents, ranging between 16 and 26 [16,17].

### Survey Creation

The survey underwent dynamic iterative review (VN, YS-B, and RR) to optimize its design, alongside the adaptation of previously validated surveys: the System Usability Scale (SUS), a simple 5-point Likert scale (eg, “strongly disagree,” “disagree,” “neither agree nor disagree,” “agree,” and “strongly agree”) used to measure subjective assessments of usability, and the mHealth App Usability Questionnaire (MAUQ), a questionnaire developed from existing validated surveys to assess the usability of a novel health care smartphone app [18,19]. The final survey (Multimedia Appendix 1) used for our project was nonvalidated, but its components have been previously used in the ophthalmology field [20]. There is evidence to suggest acceptable reliability across a range of usability scales, from single to multi-item measures, suggesting that the underlying constructs are robustly captured even when item sets are modified for contextual fit [21].

The statements were adapted from the MAUQ to focus on the image-capture workflow rather than a smartphone app, assessing 3 domains: ease of use, interface and satisfaction, and usefulness. Statements 1 to 2 of the survey covered “ease of use,” 3 to 5 addressed “interface and satisfaction,” and 6 to 7 focused on “usefulness.” The SUS and MAUQ were not applied as stand-alone validated instruments in their original scored form; instead, only selected items served as a conceptual source for usability framing and descriptive statistics. The use of single-item usability measures has been demonstrated to be equally reliable as more substantially itemed questionnaires [21,22].

A 5-point Likert scale, adapted from the SUS, was included to provide a numerical measure for each subjective question, ranging from 1 to 5. An open-text box allowed for additional reflective comments from survey participants. The same survey was presented for each workflow. Price ranges were provided for each workflow to inform the response to statement 7, referring to cost-effectiveness. The survey also collected participants’ staff grades while maintaining full anonymity. Finally, a question at the end was provided to invite participants to opt into a semistructured interview for further discussion and reflection on ASP workflows.

### Data Collection

Surveys were disseminated within a single center. Participants were introduced to the project aims and given a brief tutorial on workflow operations to ensure consistent understanding, alongside a familiarity exercise. The evaluation focused on user experiences, both prior and novel, to provide a qualitative, quality-improvement perspective. Each workflow was trialed synchronously before survey completion, supporting experiential comparisons. Following survey completion, one-on-one interviews were conducted face-to-face or virtually via Zoom using an interview guide (Multimedia Appendix 2), with verbal consent obtained for audio recording. Zoom interviews were offered to accommodate clinicians’ schedules rather than to selectively recruit technology-savvy participants.

To extend the analysis beyond usability and user preferences, the potential clinical applicability of ASP workflows was explored. This is particularly important given that many BYOD and ASP systems are increasingly designed not only for image acquisition but also to support clinical decision-making. Therefore, our project question in this regard was to ascertain what the clinical applications of these ASP workflows were with respect to clinical decision-making, specifically in informing diagnostic and monitoring decisions.

**Figure 2.**
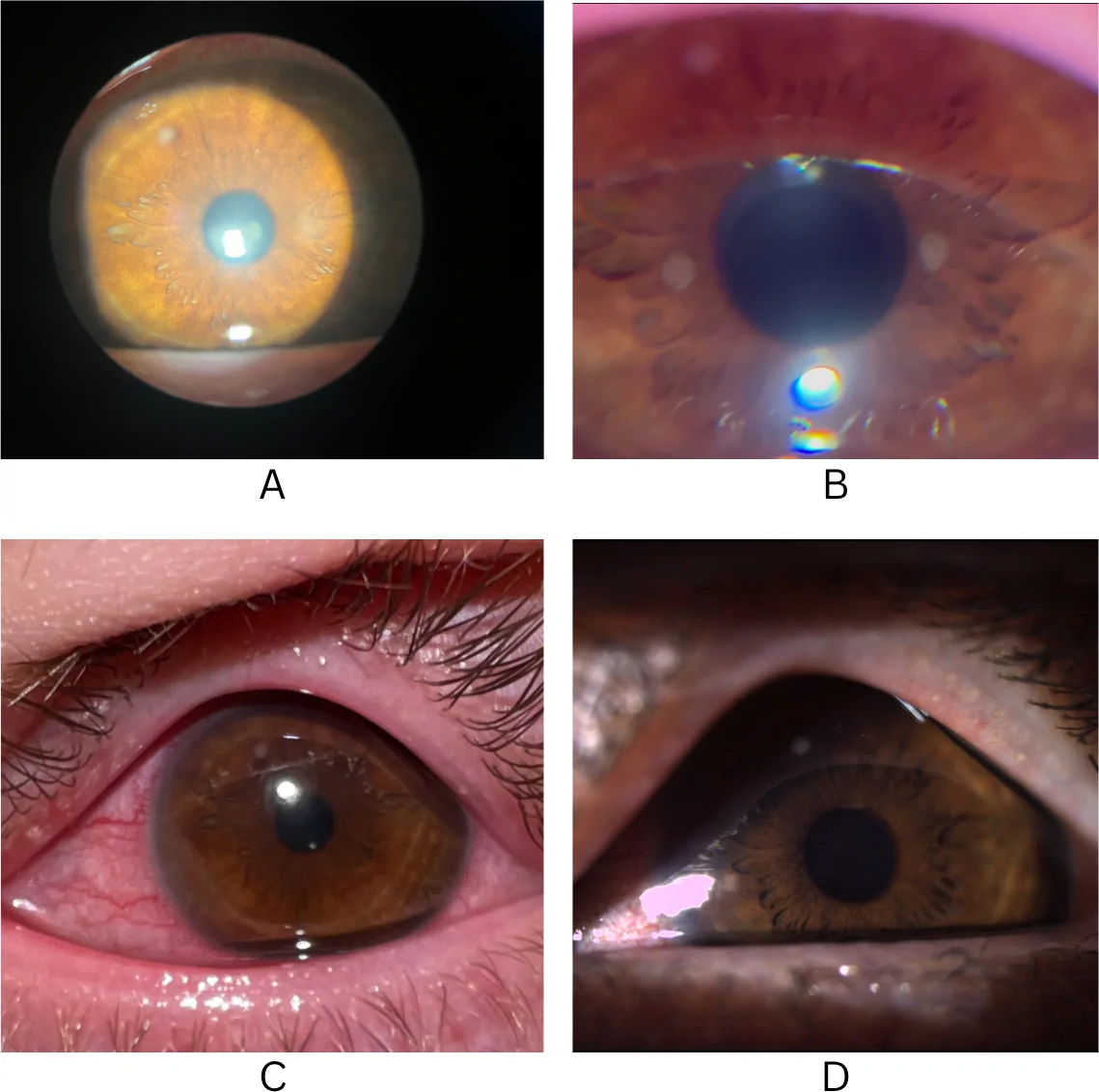
A 21-year-old contact lens wearer presents to accident and emergency with pain and a red eye after sleeping in contact lenses overnight. These were the 4 clinical images that were available to project participants, each taken using a separate anterior segment photography (ASP) workflow and a clinical vignette: (A) NexYZ, (B) QuikVue, (C) Magnifier app, and (D) Zeiss Slit Lamp Imaging Solution. Image consent was obtained from the patient for research and project dissemination.

At the end of each interview, respondents were presented with a brief clinical case involving microbial keratitis, a common ophthalmic condition with potentially sight-threatening consequences if misdiagnosed or inadequately monitored. The condition was deliberately selected as a straightforward and common clinical scenario in which image quality may directly influence diagnostic confidence and longitudinal assessment. Participants were shown 4 unlabeled images of the same patient’s eye, each captured using a different ASP workflow (Figure 2). Due to the visual nature of the ophthalmology specialty, diagnostic images were presented on printed A4 paper, and no additional clinical information or prompting was provided beyond the vignette.

Clinicians were asked to comment on image characteristics, identify relevant pathology, compare image quality between workflows, and match each image to its perceived source before answers were revealed. This exercise was designed to assess whether images obtained using different ASP workflows could provide sufficient information to support clinically meaningful decisions. With respect to the clinical utility scoring, participants were asked whether each image was adequate to inform a diagnostic or monitoring decision independently, or whether a conventional slit-lamp examination would still be required. This approach allowed the evaluation of the potential role of ASP workflows in supporting real-world clinical pathways beyond image capture alone.

### Data Analysis

The survey results were collated and analyzed in Microsoft Excel, and interviews were transcribed using Otter.ai and subsequently reviewed for accuracy by the authors (VN and YS-B). Consistent with the exploratory aims of this project, survey data were summarized descriptively; mean (SD) Likert-scale responses were calculated for each item, used to contextualize qualitative findings, and presented in a table. Additional comments from free-text boxes were thematically analyzed alongside interview transcripts using NVivo (Lumivero).

The use of means and SDs to summarize Likert-scale responses is a widely debated but commonly accepted practice in exploratory mixed methods studies [23,24]. Given the exploratory and quality improvement framing of our project, mean Likert-scale scores were intended as descriptive summaries to contextualize qualitative findings rather than as inferential statistics. These values summarize clinician perceptions of the complete imaging workflows and should be read as descriptive only. They are influenced by confounding factors, such as smartphone familiarity, capture software, and prior experience with BYOD governance, and are not intended to support statistical comparisons between ASP workflows. This is particularly relevant for the “intention to use again” domain, which is shaped by a clinician’s existing familiarity with and acceptance of ASP rather than the imaging workflow alone. Formal hypothesis testing with statistical analysis was outside the scope of this evaluation, given the exploratory design and sample size.

The authorship team undertook a reflexive exercise prior to interviews, in which personal assumptions and positionalities were explicitly acknowledged as an active part of the analytical process; a predominantly constructivist research paradigm was adopted for the mixed methods project. All transcripts and comments were coded by a single author (VN); codes were iteratively developed into candidate subthemes, with those sharing analytical constructs consolidated into themes. This coding was then critically reviewed and interrogated by 2 authors (YS-B and RR) acting as “critical friends,” using an online mind-mapping process (Canva) to challenge and refine the developing themes rather than establishing intercoder agreement. This generative process of codes, subthemes, and themes reflects the interpretive flexibility of reflexive thematic analysis, wherein analytic constructs are understood to be author-generated and subject to human bias, rather than an inherent property of the data [25,26]. The thematic analysis reflects the mixed methods nature of our project, where interpretation of data is subjective, adopting a constructivist research paradigm.

The quantitative and qualitative components were analyzed and reported separately in the *Results* section. Likert-scale responses were summarized descriptively and were not incorporated into the thematic analysis. The only exception was that free-text survey comments were pooled with the interview transcripts and coded as a single qualitative dataset, so that both sources contributed to the same subthemes and themes. Descriptive Likert-scale ratings and qualitative themes were considered together at the discussion stage, where consistency between them supported an interpretation, and discrepancies were retained as reflecting variation in clinician experience.

## Results

### Participant Recruitment and Interview Characteristics

A total of 35 residents and fellows were approached over a 4-week period, with 25 survey responses collected: 17 (68%) ophthalmology residents and 8 (32%) corneal fellows; 10 participants declined to participate due to timing constraints. Of this cohort, 16 expressed interest in an interview, and 12 interviews were completed (25% drop-out rate). The mean interview duration was 18.1 (SD 10.8) minutes.

### Survey Results

The survey data were first charted by collating Likert scale responses for each ASP workflow. Mean Likert scale response scores were calculated for each question and imaging workflow and are presented in Table 1. Clinician perceptions favored the Magnifier workflow, which received the highest ratings for usability, cost-effectiveness, and clinical utility (19/25, 76%, 23/25, 92%, and 18/25, 72% strongly agreed, respectively), with consistently favorable ratings across the evaluated domains.

**Table 1.** Mean Likert scale ratings of 4 anterior segment photography (ASP) workflows across 7 perception domains^a^^,^^b^.

ASP workflow	Ease of use, mean (SD)	Ease of learning, mean (SD)	Design appeal, mean (SD)	Time efficiency, mean (SD)	Intention to use again, mean (SD)	Clinical utility, mean (SD)	Cost-effectiveness, mean (SD)
Magnifier	4.68 (0.69)	4.72 (0.44)	4.44 (0.65)	4.72 (0.54)	4.44 (0.92)	4.68 (0.56)	4.92 (0.28)
QuikVue	4.28 (0.79)	4.44 (0.77)	4.16 (1.11)	4.16 (0.94)	4.16 (1.21)	4.48 (0.71)	2.96 (1.14)
NexYZ	3.28 (1.06)	3.92 (0.70)	3.20 (0.91)	3.24 (1.01)	3.28 (1.06)	3.88 (0.73)	4.12 (0.93)
ZSLIS^c^	3.88 (0.78)	3.92 (0.91)	4.28 (0.68)	3.92 (0.91)	4.32 (0.85)	4.56 (0.58)	2.36 (1.08)

a Values represented in this table are descriptive summaries of clinician perceptions of the complete imaging workflows and are not intended for statistical comparison between workflows, nor are they adjusted for confounding factors including workflow type, user familiarity, software, hardware adapters, and information governance.

b Values presented as mean on a 5-point Likert scale, where higher scores indicate more favorable perceptions.

c ZSLIS: Zeiss Slit Lamp Imaging Solution.

### Thematic Analysis

#### Theme 1: Obstacles to Clinical Implementation

The most frequently referenced theme was “obstacles to clinical implementation,” which encompassed challenges in integrating ASP workflows into practice. Although clinicians recognized the clinical value of these images, they consistently reported barriers, including design flaws, lengthy workflows, and limited accessibility.

Workflow limitations were a recurrent concern. The Magnifier workflow was constrained by smartphone camera quality and the absence of a cobalt-blue filter; the QuikVue workflow lacked a slit-beam, limiting full AS assessment. Clinically important features—slit-beam illumination, wide-angle view, and adequate magnification—were often inadequate. Design issues added to frustration: the QuikVue workflow was incompatible with some smartphones and prone to image distortion due to the nature of its native lens, while the NexYZ workflow was described as “bulky” and “fiddly,” reflecting its nonmedical design and time-consuming workflow to attach to a smartphone, followed by a slit-lamp. The ZSLIS workflow, too, presented difficulties, including a steep learning curve and suboptimal synchronization between its optical system and camera. Accessibility compounded these issues, with difficulties in sourcing and reliably locating the NexYZ and QuikVue, both of which were viewed as easily misplaced or borrowed by other departments when not attached to a smartphone, while the ZSLIS was considered prohibitively expensive and not readily available in many units.

Finally, a sense of discouragement emerged. Clinicians reported a reluctance to use ASP despite recognizing its benefits, citing practical irritations such as removing phone cases for the QuikVue workflow, the disproportionate setup time of the NexYZ workflow for a single image, and ZSLIS-related workflow disruption, which often required moving patients and transferring images through a separate computer system.

Taken together, these challenges created frustration that impeded widespread uptake. Of the workflows, the ZSLIS workflow posed the greatest obstacles, including high cost, lack of portability, workflow disruption, and training requirements, followed by the NexYZ workflow, which was criticized for its bulk and time-consuming setup. The QuikVue workflow faced issues of compatibility and limited functionality, while the Magnifier workflow, though restricted by smartphone quality and lacking a cobalt-blue filter, was viewed as presenting fewer barriers overall.

#### Theme 2: Accessibility

Theme 2, “accessibility,” highlighted each workflow’s positive attributes and potential to enhance practice, with 3 subthemes: ease of use, portability, and design. The Magnifier workflow was most praised, particularly in eye casualty, where equipment is often misplaced. Its integration with clinicians’ own smartphones removed the need for additional tools, making it suitable for wards and patients with limited mobility. Respondents valued its simplicity and speed. It was also effective in complex scenarios, such as pediatric cases, aided by rapid shutter speeds, although ultimately dependent on the smartphone.

The NexYZ workflow was commended for stability; once assembled, it required minimal adjustment for consistent imaging. Portability was another strength, with the Magnifier and QuikVue workflows frequently noted for compactness. The QuikVue workflow was described as “probably the most portable,” requiring no slit-lamp and easily attachable to smartphones for opportunistic imaging. Design features were also valued: the QuikVue workflow’s built-in cobalt-blue light and the NexYZ workflow’s ability to use slit-lamp optics supported detailed corneal assessment, offsetting earlier design criticisms.

Overall, the Magnifier workflow ranked highest for accessibility due to smartphone compatibility, ease of use, and versatility across settings. The QuikVue workflow followed, valued for portability and cobalt-blue light; the NexYZ workflow offered stability but required a slit-lamp and greater workflow time. The ZSLIS workflow ranked lowest, constrained by cost, bulk, and training demands.

#### Theme 3: Quality and Workflow

Theme 3, “quality and workflow,” captures clinicians’ perspectives on ASP, focusing on image quality, uploading, and standardization. The ZSLIS workflow was consistently rated highest for image quality, particularly in monitoring slowly progressing conditions such as iris nevi or fungal infections, owing to its superior resolution and sophisticated camera optics.

Image upload proved problematic across all workflows. Smartphone-based uploads, the Magnifier, QuikVue, and NexYZ workflows, were viewed as inefficient and prone to delays due to login or connectivity issues, while the ZSLIS workflow’s reliance on the proprietary Zeiss database often hindered image extraction. Image capture standardization was deemed essential for diagnostic consistency, follow-up, research, and AI use. Furthermore, the QuikVue and Magnifier workflows were praised for fixed focal lengths that promote standardization, though the Magnifier’s design necessitated close proximity to the patient, sometimes causing discomfort.

For overall image capture, the ZSLIS workflow ranked highest for quality and clinical utility, followed by the NexYZ workflow for slit-lamp compatibility. The QuikVue workflow was ranked third for its standardization benefits and built-in lighting, while the Magnifier workflow ranked lowest, offering portability and ease of use at the cost of image quality.

#### Theme 4: Digital Ethics

The use of personal devices introduces considerations for data protection and information governance (IG). Theme 4, “digital ethics,” explores the ethical and practical challenges associated with using personal devices in clinical settings, focusing on 2 subthemes: data protection and personal device use.

Respondents raised concerns about potential breaches of patient confidentiality when personal devices are used in clinical practice, particularly when connected to unsecured networks or when delays in uploading images introduce the risk of human error, with 1 participant detailing, “the issue of, if you are using your own device, where are the images being saved?” Participants highlighted a perceived tension between the convenience of digitally connected devices and confidence in the security of captured images. Notably, these concerns persisted despite the availability of established BYOD infrastructure and IG-compliant image-sharing solutions within the trust. Several respondents expressed uncertainty regarding image storage pathways, cloud connectivity, and the practical application of local BYOD policies, suggesting that a lack of governance awareness may represent a barrier to adoption independent of the governance frameworks themselves. This finding highlights that successful implementation of smartphone-based ASP workflows may require not only robust governance systems but also clinician understanding and confidence in those systems.

The use of personal devices further raised issues relating to professionalism, infection control, and standardization, given the variability in device types and clinician preferences. Moreover, patient perceptions of clinicians using personal phones for clinical photography were identified as a potentially sensitive issue. Although MEH offers access to Pando, an IG-compliant platform for secure image sharing, these broader ethical and procedural considerations highlight the complexity of integrating personal technology into routine clinical practice.

MEH has its own distinct BYOD network to facilitate BYOD photography, with dedicated EPR integration. The views expressed by respondents, therefore, appear to reflect uncertainty surrounding the practical implementation and understanding of BYOD governance, rather than deficiencies in the trust’s existing governance infrastructure.

#### Theme 5: Innovation

Theme 5, “innovation,” captures clinicians’ aspirations for advancing ASP through streamlined, purpose-built solutions. Respondents expressed a desire for an app that fully uses the smartphone’s camera features, avoids saving images to the device’s camera roll, and automatically uploads them to the EPR, thereby enhancing efficiency and maintaining IG compliance. Among those who did not mention an all-in-one application, many discussed the idea of an all-in-one slit-lamp, integrated with immediate photography and uploading capabilities that maintain IG compliance and image quality.

Together, these ideas reflect a clear clinician desire for image capture technologies that simplify clinical workflows while improving on current clinical standards.

**Figure 3.**
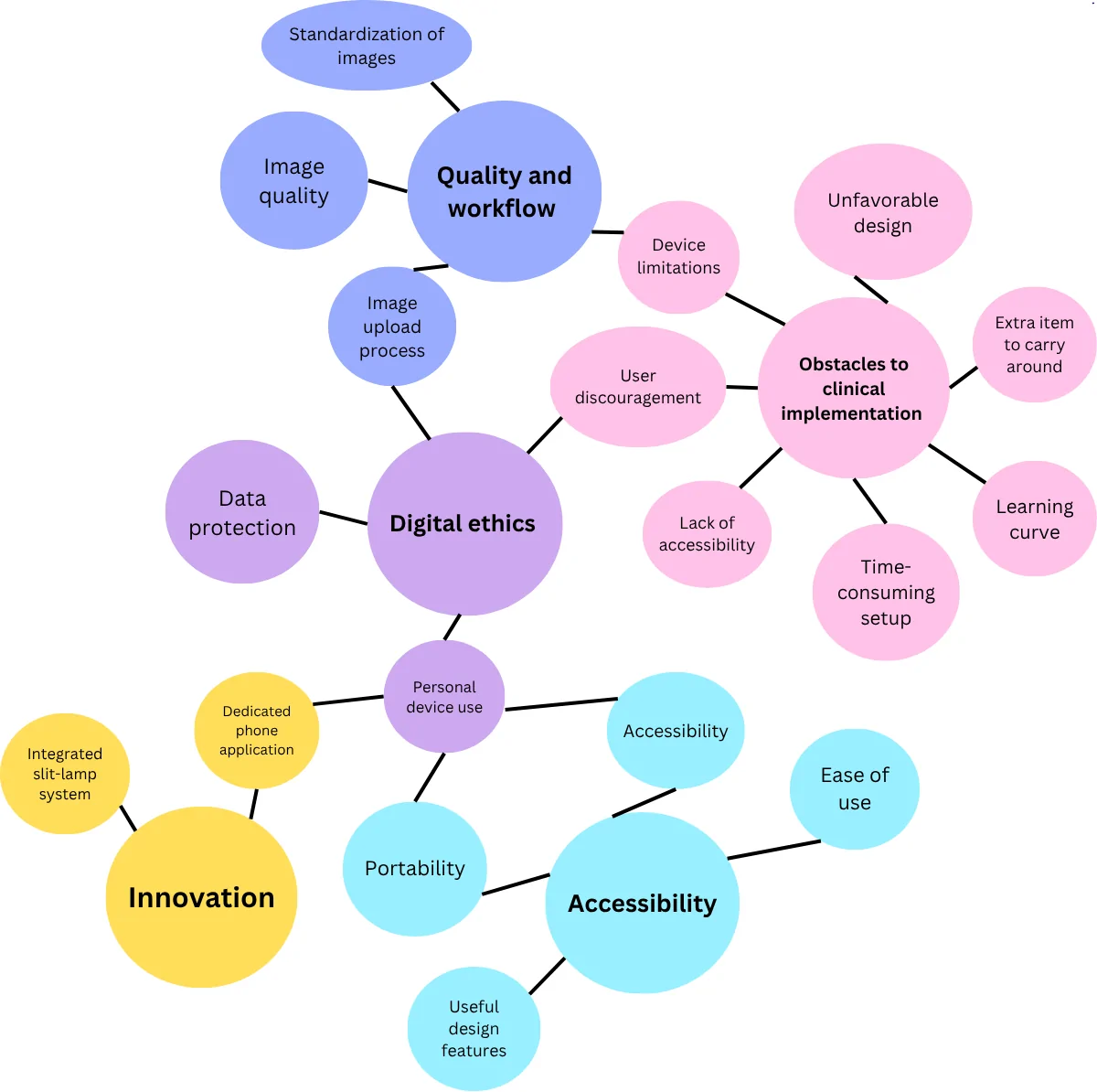
A map chart detailing the 5 key themes (obstacles to clinical implementation, accessibility, quality and workflow, digital ethics, and innovation) that arose from thematic analysis of interview transcripts and free-text comments from surveys, and the subthemes that are interconnected.

A summary map of the thematic analysis results, a visual representation of the emerging subthemes and how they are collapsed into themes, can be found in Figure 3.

### Additional Findings

Interview respondents were asked about their preferred ASP workflow in the context of clinical utility, assessed through a review of captured images from a clinical case of microbial keratitis. The respondents’ preferences also included a subjective summation of usability aspects, as explored through thematic analysis and surveys. It was revealed that the majority preferred the Magnifier workflow. Table 2 shows overall preferences in a clinical utility context, though these findings reflect clinician-perceived clinical utility based on a single illustrative case.

**Table 2.** Ophthalmology residents and corneal fellows’ favorite anterior segment photography (ASP) workflows by percentage in the context of clinical utility when presented with images of a microbial keratitis case (N=12).

ASP workflow	Respondents expressing preference, n (%)
Magnifier	5 (42)
QuikVue	2 (17)
NexYZ	3 (25)
ZSLIS^a^	2 (17)

a ZSLIS: Zeiss Slit Lamp Imaging Solution.

### Clinical Case Discussion

When presented with images of a corneal infiltrate from the clinical case of microbial keratitis, all respondents identified the correct clinical diagnosis and localized it to the appropriate region of the cornea. Across workflows, variation was observed in global image characteristics such as field of view, depth of field, resolution, illumination, and the presence of reflections. For example, the Magnifier image encompassed the entire globe, allowing appreciation of conjunctival injection in addition to a faint outline of the infiltrate. The QuikVue image demonstrated higher magnification, but was limited by prominent reflections, reducing structural visibility. The NexYZ image, while also highly magnified and lacking conjunctival context, conveyed greater information about the depth of the lesion. The ZSLIS image offered the highest resolution, with fine adnexal detail, though uneven illumination introduced shadowing at the lesion site.

When explored in interviews, clinician preferences reflected these differing characteristics: the NexYZ workflow was selected as the most informative for diagnostic purposes (n=5, 42%), while the Magnifier workflow was most frequently identified as the second most useful (n=4, 33%). It should be emphasized that these results highlight a preference for the ASP workflow in the context of producing a diagnostically informative image during interviews, rather than an objective diagnostic accuracy assessment.

## Discussion

This mixed methods evaluation explored 4 ASP workflows and clinicians’ perceptions of their clinical utility. Clinicians highlighted the value of ASP in documentation, referrals, and monitoring, though no single workflow emerged as universally optimal. This likely reflects that the clinical context and the purpose of image acquisition influence which ASP approach is most appropriate on a case-by-case basis. The Magnifier workflow scored highest for usability, clinical utility, and cost-effectiveness. The QuikVue workflow was rated positively for usability but criticized for its cost, incompatibility with certain smartphone models, and challenges in capturing certain pathologies. The NexYZ and ZSLIS workflows received mixed feedback, with some concerns about usability, although their contribution to clinical assessment was acknowledged.

There are several existing studies that explore ASP workflows with some overlapping findings with this project. Pujari et al [27] evaluated a smartphone clip-on device like QuikVue without examining clinical integration. Similarly, Aswin [28] described using a smartphone with a slit-beam adjunct for AS imaging, presenting a different approach that could be combined with the Magnifier app. A study by Mamtora et al [4] shared similar objectives to this project, where there was a focus on a single ASP workflow (QuikVue) that could improve HES referrals to eye care services. Mamtora et al [4] captured both cobalt-blue and white-light images for different AS pathologies; their narrower research question and comparison of only one device provided valuable background for this project.

Beyond singular device-specific evaluations, our findings resonate with a growing body of work on smartphone slit-lamp imaging. In a noninferiority study, Goel et al [29] found that ASP images captured on a smartphone coupled to a slit-lamp adapter were subjectively noninferior to those from a commercial built-in slit-lamp camera, as judged across all grades of ophthalmic staff. Muth et al [30] similarly reported that a smartphone slit-lamp adapter produced high-quality AS images and was faster for image acquisition than a dedicated ophthalmic photographer, concluding that such setups offer a cost-effective, portable solution to support documentation and communication between colleagues. The usability dimension our project explores is likewise reflected in the wider literature; Emam et al [31] demonstrated ease of use in an ASP-naive medical student cohort following a brief hands-on instructional course. Our project advances this body of work in 3 respects: comparing multiple imaging workflows within a single clinical service rather than evaluating 1 adapter in isolation; embedding the evaluation in a real-world clinical example rather than controlled image-capture tasks; and pairing quantitative ratings with qualitative methods to triangulate the themes that shape ASP workflow implementation in clinical practice.

Alongside these contributions, our survey and interview data indicated barriers to adoption, particularly the administrative burden of documentation and integration with EPR systems. Evidence suggests such documentation pressures contribute to cognitive overload and burnout [32]. The Magnifier workflow’s popularity may therefore reflect its simplicity and compatibility with existing smartphone infrastructure, especially valuable in eye casualty, where rapid image capture can complement written notes. The NexYZ workflow was viewed as more useful in corneal clinics, leveraging slit-lamp optics for detailed layer-by-layer assessment using the crossbeam function and video recording, allowing for dynamic appreciation and documentation of pathology. The QuikVue workflow, though less suited to corneal pathology due to glare, was appreciated for its cobalt-blue illumination and portability, making it relevant in community or emergency settings where slit-lamp access is limited.

Evidence indicates that there is a growing movement toward telemedicine in eye health care services and that an estimated 98% of health care personnel own personal smartphones [33]. The General Medical Council (GMC) guidance on capturing clinical images emphasizes patient consent, with storage policies varying by trust [34]. This creates ambiguity where trusts lack specific policies for BYOD photography, or where variation exists in the wider health care system. Clinician interviews provided insights into IG compliance and BYOD policies, noting that the Magnifier workflow enables secure sharing by compartmentalizing images without uploading to the smartphone’s image gallery. However, a key barrier to widespread BYOD adoption may be the lack of clear guidance and organizational knowledge of approved and standardized approaches to BYOD photography. Concerns were also raised about biometric data safety, particularly as iris images are increasingly used in recognition technologies, underscoring the need for broader safety considerations [35].

Beyond immediate clinical use, respondents noted potential benefits for education, training, and AI development. By embedding images directly into referral correspondence, digital ASP could improve referral quality and efficiency, support intrahospital discussion, and facilitate team-based decision-making with minimal disruption. These findings align with wider shifts toward telemedicine and digital ophthalmology [6]. The literature shows that including AS images with referrals enhances diagnostic accuracy over written histories alone, and smartphone-acquired images are increasingly positioned within teleophthalmology pathways that connect nonspecialists or remote practitioners with tertiary corneal services through automated image triaging [4,36]. The trajectory toward AI integration and AI-assisted clinical pathways lends weight to these ASP workflows. Deep-learning models have demonstrated the ability to classify and differentiate between AS conditions, including infectious keratitis and its causative organisms, directly from smartphone-acquired images [37,38]. Notably, Maehara et al [38] used images captured on the iPhone 13 Pro, the same as in this present project, and demonstrated diagnostic accuracy comparable to slit-lamp images when interpreted with AI assistance [38]. These findings highlight the wider shift toward AI-augmented pathways in conventional telemedicine and digital ophthalmology.

This project had several limitations. First, the 4 workflows represent different ASP categories that differ in purpose, setup, portability, and workflow requirements. The findings should, therefore, be read as a real-world exploration of clinician experience rather than a head-to-head technical comparison. The ZSLIS workflow, in particular, required a separate cubicle and dedicated imaging setup, which may have shaped perceptions of accessibility and workflow integration. As an exploratory quality-improvement evaluation, the project was not designed or powered for hypothesis testing, and quantitative findings are presented descriptively.

Second, the clinical case component should be interpreted cautiously. It used a single illustrative case to contextualize clinician-perceived image utility rather than to evaluate workflow performance, and image quality in this setting may reflect both the technical properties of each workflow and how it was used on that day. We did not objectively measure diagnostic accuracy, workflow time, downstream clinical impact, or image quality against standardized grading criteria.

Finally, several sources of bias should be acknowledged. Participants varied in prior familiarity with smartphone-based photography, reflecting general rather than workflow-specific experience. Unequal familiarity with the individual components of each workflow may also have influenced perceptions despite standardized tutorials. Voluntary participation introduces possible self-selection toward clinicians with greater interest in digital imaging. All participants completed the survey immediately after hands-on use under identical conditions; however, interviews were conducted both face-to-face and via Zoom to accommodate schedules, which may have introduced response bias. Consistent with reflexive thematic analysis, coding was undertaken by a single author, with structured critical review by 2 others to mitigate sole-coder bias. The single-center setting, small sample, and resident or fellow cohort further limit generalizability to other settings and health care systems.

This evaluation focused on clinician experience in corneal and eye casualty settings. Future research should therefore evaluate integration into referral and triage pathways, formal cost analyses, comparisons between AS videography and photography, objective measures of image quality and workflow efficiency, and the impact of ASP on clinical decision-making and patient outcomes. Future studies should also focus on using standardized acquisition protocols, masked grading, timed workflow assessment, and an appropriate diagnostic reference standard. Patient perceptions of smartphone use in clinical settings also require attention. While smartphone photography offers clear benefits, some patients may perceive it as unprofessional [39]. Further research is necessary to explore patient views on ophthalmic photography and its implications for clinical practice.

In an era of expanding telemedicine, smartphone use, and AI-based decision support tools, ASP is likely to play an increasing role in clinical workflows. Within corneal and eye casualty settings at a single tertiary center, this evaluation did not identify a single optimal ASP workflow. Most respondents expressed a preference for the Magnifier workflow, which may reflect its ease of use, interface, cost, and integration with existing smartphone infrastructure. Our findings suggest that dedicated guidelines, documentation systems, and educational resources may help clinicians capture standardized images while safeguarding patient data, though this warrants further evaluation. Finally, understanding patient perspectives remains critical for ensuring that digital ASP enhances, rather than undermines, the quality of ophthalmic care.

## Supplementary material

10.2196/89441 Multimedia Appendix 1 The survey, which was disseminated to participants, exploring their perceptions of the 4 devices tested.

10.2196/89441 Multimedia Appendix 2 The interview guide used in all interviews to allow for consistency.

## Abbreviations

AS: anterior segment
ASP: anterior segment photography
BYOD: Bring Your Own Device
EPR: electronic patient record
GDPR: General Data Protection Regulation
GMC: General Medical Council
HES: Hospital Eye Services
IG: information governance
MAUQ: mHealth App Usability Questionnaire
MEH: Moorfields Eye Hospital
NHS: National Health Service
QIP: quality-improvement project
SUS: System Usability Scale
ZSLIS: Zeiss Slit Lamp Imaging Solution
