# Supplementary material for: Digital Anterior Segment Photography in Clinical Practice: Mixed Methods Study
Source: JMIR Form Res. 2026 Sep 4;10:e89441. doi: 10.2196/89441 (PMC13544412; doi:10.2196/89441)
Supplement: Supplementary file 1 [file formative-v10-e89441-s001.docx]

This survey aims to collate opinions about four different corneal photography modalities.

Regarding manually taking a photograph using a mobile phone plus app (for example, Magnifier)

|  | Strongly disagree | Disagree | Neither agree nor disagree | Agree | Strongly agree |
| --- | --- | --- | --- | --- | --- |
| 1. This device is easy to use | 1 | 2 | 3 | 4 | 5 |
| 2. It was easy for me to learn to use the device | 1 | 2 | 3 | 4 | 5 |
| 3. I like the design of the device | 1 | 2 | 3 | 4 | 5 |
| 4. The amount of time using the device is fitting for me | 1 | 2 | 3 | 4 | 5 |
| 5. I would use this device again | 1 | 2 | 3 | 4 | 5 |
| 6. This device would be useful in clinical practice | 1 | 2 | 3 | 4 | 5 |
| 7. This device is cost-effective | 1 | 2 | 3 | 4 | 5 |

Any additional comments:

Regarding an adaptor that can be mounted onto a mobile phone (for example, QuikVue)

(Photo of QuikVue, image credit <https://www.graftonoptical.com/product-page/quikvue-plus-smartphone-imaging-adaptor>)

|  | Strongly disagree | Disagree | Neither agree nor disagree | Agree | Strongly agree |
| --- | --- | --- | --- | --- | --- |
| 1. This device is easy to use | 1 | 2 | 3 | 4 | 5 |
| 2. It was easy for me to learn to use the device | 1 | 2 | 3 | 4 | 5 |
| 3. I like the design of the device | 1 | 2 | 3 | 4 | 5 |
| 4. The amount of time using the device is fitting for me | 1 | 2 | 3 | 4 | 5 |
| 5. I would use this device again | 1 | 2 | 3 | 4 | 5 |
| 6. This device would be useful in clinical practice | 1 | 2 | 3 | 4 | 5 |
| 7. This device is cost-effective | 1 | 2 | 3 | 4 | 5 |

Any additional comments:

Regarding an adaptor which can be mounted onto a mobile phone that can allow a mobile phone to capture an image from a slit lamp (for example, NeXYZ)

(Photo of NeXYZ, image credit <https://www.celestron.com/products/nexyz-3-axis-universal-smartphone-adapter>)

|  | Strongly disagree | Disagree | Neither agree nor disagree | Agree | Strongly agree |
| --- | --- | --- | --- | --- | --- |
| 1. This device is easy to use | 1 | 2 | 3 | 4 | 5 |
| 2. It was easy for me to learn to use the device | 1 | 2 | 3 | 4 | 5 |
| 3. I like the design of the device | 1 | 2 | 3 | 4 | 5 |
| 4. The amount of time using the device is fitting for me | 1 | 2 | 3 | 4 | 5 |
| 5. I would use this device again | 1 | 2 | 3 | 4 | 5 |
| 6. This device would be useful in clinical practice | 1 | 2 | 3 | 4 | 5 |
| 7. This device is cost-effective | 1 | 2 | 3 | 4 | 5 |

Any additional comments:

Regarding an integrated slit lamp camera (for example, Zeiss SL Imaging Solution)

(Photo of Zeiss SL Imaging Solution, image credit https://www.zeiss.com/meditec/en/products/basic-diagnostic-devices/zeiss-slit-lamps/sl-800.html)

|  | Strongly disagree | Disagree | Neither agree nor disagree | Agree | Strongly agree |
| --- | --- | --- | --- | --- | --- |
| 1. This device is easy to use | 1 | 2 | 3 | 4 | 5 |
| 2. It was easy for me to learn to use the device | 1 | 2 | 3 | 4 | 5 |
| 3. I like the design of the device | 1 | 2 | 3 | 4 | 5 |
| 4. The amount of time using the device is fitting for me | 1 | 2 | 3 | 4 | 5 |
| 5. I would use this device again | 1 | 2 | 3 | 4 | 5 |
| 6. This device would be useful in clinical practice | 1 | 2 | 3 | 4 | 5 |
| 7. This device is cost-effective | 1 | 2 | 3 | 4 | 5 |

Any additional comments:

For reference:

QuikVue - £250-300

NeXYZ - £60-120

Zeiss SL – £10k-20k

Name:

Grade:

Would you like to participate in a brief (20 minutes) semi-structured interview? (please circle)

Yes	No

If yes, please leave a contactable email address.

Any questions or queries, please do not hesitate to contact Vidhi Naik by email – [v.naik.20@abdn.ac.uk](mailto:v.naik.20@abdn.ac.uk)
