# Supplementary material for: Digital Anterior Segment Photography in Clinical Practice: Mixed Methods Study
Source: JMIR Form Res. 2026 Sep 4;10:e89441. doi: 10.2196/89441 (PMC13544412; doi:10.2196/89441)
Supplement: Supplementary file 2 [file formative-v10-e89441-s002.docx]

**Interview Questions**

*Regarding using a mobile phone and app to capture an image (for example, Magnifier)*

*Regarding an adaptor that can be mounted onto a mobile phone (for example, QuikVue)*

*Regarding an adaptor which can be mounted onto a slit lamp that can allow a mobile phone to capture an image from a slit lamp (for example, NeXYZ)*

*Regarding an integrated slit lamp camera (for example, Zeiss SL 800)*

| No. | Question |
| --- | --- |
| 1 | What do you think are the benefits of using each imaging modality? |
| 2 | What do you think the disadvantages/limitations are? |
| 3 | Is this something you would want to use in Moorfields’ clinical practice, and if so, why/why not? |
| 4 | If you could choose one to be made widely available – which one would you choose? (which is your favourite) |
| 5 | What do you think of the current process of taking corneal photographs? |
| 6 | What do you think of the image access/upload process for corneal photos? |
| 7 | How confident would you feel on a scale of 1-5 (1 not confident to 5 very confident) in making a clinical decision based on history vs history + image from each modality in the context of…   - Making a clinical decision on whether to accept a referral from the community (i.e., "no referral", "emergency referral", "urgent referral" or "routine referral") - Suggesting a management plan if advising a junior ophthalmologist or A+E practitioner |
| 8 | What would be the ideal pipeline for capturing clinical photographs and uploading them to the patient records? |

**Clinical Case and Image Questions**

(four images from each modality of the same patient with a corneal pathology)

Clinical case: 21 y/o contact lens wearer presents with pain and a red eye after sleeping in contact lenses overnight

| No. | Question |
| --- | --- |
| 1 | What is the diagnosis? |
| 2 | From which image was it easiest to ascertain the diagnosis? |
| 3 | Which image has the best quality, in your opinion? |
